# Pharmacological activation of autophagy favors the clearing of intracellular aggregates of misfolded prion protein peptide to prevent neuronal death

**DOI:** 10.1038/s41419-017-0252-8

**Published:** 2018-02-07

**Authors:** Stefano Thellung, Beatrice Scoti, Alessandro Corsaro, Valentina Villa, Mario Nizzari, Maria Cristina Gagliani, Carola Porcile, Claudio Russo, Aldo Pagano, Carlo Tacchetti, Katia Cortese, Tullio Florio

**Affiliations:** 1 Section of Pharmacology, Department of Internal Medicine (DiMI), and Centre of Excellence for Biomedical Research (CEBR), University of Genova, Genova, Italy; 2 Section of Human Anatomy, Department of Experimental Medicine (DIMES), School of Medicine, University of Genova, Genova, Italy; 3 Department of Health Sciences, University of Molise, Campobasso, Italy; 4 Ospedale Policlinico San Martino, IRCCS per l’Oncologia, Genova, Italy; 5 Centro Imaging Sperimentale, IRCCS Istituto Scientifico San Raffaele, Milano, Italy; 6 Vita-Salute San Raffaele University, Milano, Italy

## Abstract

According to the “gain-of-toxicity mechanism”, neuronal loss during cerebral proteinopathies is caused by accumulation of aggregation-prone conformers of misfolded cellular proteins, although it is still debated which aggregation state actually corresponds to the neurotoxic entity. Autophagy, originally described as a variant of programmed cell death, is now emerging as a crucial mechanism for cell survival in response to a variety of cell stressors, including nutrient deprivation, damage of cytoplasmic organelles, or accumulation of misfolded proteins. Impairment of autophagic flux in neurons often associates with neurodegeneration during cerebral amyloidosis, suggesting a role in clearing neurons from aggregation-prone misfolded proteins. Thus, autophagy may represent a target for innovative therapies. In this work, we show that alterations of autophagy progression occur in neurons following in vitro exposure to the amyloidogenic and neurotoxic prion protein-derived peptide PrP90-231. We report that the increase of autophagic flux represents a strategy adopted by neurons to survive the intracellular accumulation of misfolded PrP90-231. In particular, PrP90-231 internalization in A1 murine mesencephalic neurons occurs in acidic structures, showing electron microscopy hallmarks of autophagosomes and autophagolysosomes. However, these structures do not undergo resolution and accumulate in cytosol, suggesting that, in the presence of PrP90-231, autophagy is activated but its progression is impaired; the inability to clear PrP90-231 via autophagy induces cytotoxicity, causing impairment of lysosomal integrity and cytosolic diffusion of hydrolytic enzymes. Conversely, the induction of autophagy by pharmacological  blockade of mTOR kinase or trophic factor deprivation restored autophagy resolution, reducing intracellular PrP90-231 accumulation and neuronal death. Taken together, these data indicate that PrP90-231 internalization induces an autophagic defensive response in A1 neurons, although incomplete and insufficient to grant survival; the pharmacological enhancement of this process exerts neuroprotection favoring the clearing of the internalized peptide and could represents a promising neuroprotective tool for neurodegenerative proteinopathies.

## Introduction

Protein misfolding is the main pathogenic event responsible for synaptic loss, neuronal death, and gliosis during all neurodegenerative disorders^1–4^. In particular, β-amyloid (Aβ) peptides, which are organized as insoluble amorphous aggregates or amyloid fibrils, and plaques accumulate in Alzheimer's disease (AD) patient brains^5^, while pathognomonic for Parkinson's disease (PD) is intraneuronal aggregates of α-synuclein^6^. Prion diseases (transmissible spongiform encephalopathies, TSE) are recognized with the pathogenetic mechanism of protein misfolding of the prion protein (PrP), which shifts from a physiological conformation (PrP^C^) into a protease-resistant, amyloidogenic isoform, named PrP “scrapie” (PrP^Sc^)^7–9^. In TSE patients, PrP^Sc^ oligomeric aggregates have been identified in brain areas displaying neuron degeneration, vacuolization, and glial activation^10–12^, suggesting that during PrP^C^ = > PrP^Sc^ transition neurotoxic species are generated before amyloid fibrillogenesis^13–16^. Similarly, during AD-associated neurodegeneration, Aβ1–42 is recovered as intraneuronal aggregates in brain areas where neuronal death is particularly significant^17^, anticipating amyloid deposition^18^. It was proposed that the state of aggregation of all misfolded peptides determines their neurotoxicity, oligomers being more effective in reducing neuron viability in vitro^19,20^ and cognitive ability in vivo^21,22^. Thus, neurotoxicity seems independent from the nature or function of native proteins, but caused by the increased hydrophobicity of oligomers^23–25^. In this view, all amyloidogenic polypeptides (PrP^Sc^, Aβ, and α-synuclein) share common neurodegenerative mechanisms^3,26^, via oligomers’ interaction with neuronal targets^22,27–29^, making protein misfolding and aggregation process a valuable target for disease-modifying therapies against neurodegenerative proteinopathies^30^.

Eukaryotic cells perform a tight quality control of protein synthesis to eliminate misfolded proteins^2^. Ubiquitin-proteasome and lysosome-mediated autophagy are the most relevant protein homeostasis systems^31^. Originally described as mechanism of programmed cell death, autophagy is a crucial strategy to overcome harmful conditions by recycling nutrient material. Double-membrane vesicles (autophagosomes) engulf altered proteins and organelles before receiving the hydrolytic contribution of lysosomes, thereby assuming the name of autolysosomes^32^. Autolysosome content is then digested and recycled in the cytoplasm through active transport. Thus, autophagy may prevent cell death during nutrient deprivation by allowing recycling of proteins until the energetic supply is restored^33^. Autophagosomes can also digest damaged mitochondria or aggregated proteins^34,35^, which is a critical activitiy to prevent lysosomal- and mitochondrial-dependent apoptosis, and to reduce the intracellular burden of unwanted protein^36,37^. Indeed, impairment of autophagy causes neuronal death during normal aging, AD, PD, and TSEs^38–41^. Pharmacological induction of autophagy by rapamycin, valproic acid, or lithium, favors the degradation of aggregation-prone proteins, delaying the clinical onset or reducing the symptoms in animal models of proteinopathies^41,42^.

We report the role of autophagy as a response to the neurotoxic effects of intracellular aggregation of a misfolded prion peptide, evaluating autophagy enhancers as neuroprotective agents. PrP^Sc^ neurotoxicity was modeled using a recombinant peptide matching the 90–231 sequence of human PrP^C^^43^, which corresponds to a protease-insensitive C-terminal fragment of PrP^Sc^ detected in TSE patients^44^. By controlled thermal denaturation, PrP90-231 β-sheet content increases (up to 58.8%)^45^ through interferences with determinants of helix formation^46^. β-sheet-rich PrP90-231, showing high hydrophobicity, and resistance to proteolysis, is organized as soluble oligomers, which, after prolonged denaturation, become insoluble and, eventually, amyloid fibrils^47^. PrP90-231 oligomers are neurotoxic in vitro^45,47^ with similar efficacy than brain-purified PrP^Sc^^48,49^. PrP90-231 neurotoxicity is dependent on intracellular accumulation, as insoluble aggregates cause lysosome destabilization^50,51^ and cytosolic diffusion of cathepsin D (Cat-D)^51^.

The vesicular accumulation of aggregated PrP90-231 poses the following questions about the finalistic role of its intracellular partition: (1) is it cause of cell death through the impairment of lysosomal-mediated protein homeostasis (2) or does it represent a cell attempt to neutralize the peptide through autophagic proteolysis?

Here we demonstrate that PrP90-231 increases the formation of PrP90-231-containing autophagolysosomes, but the increased autophagy does not proceed toward resolution and does not prevent neuronal death; conversely, pharmacological activation of autophagy favors aggregate clearing and counteracted PrP90-231 toxicity. Thus autophagy represents a neuronal defensive strategy against intracellular aggregation of misfolded PrP90-231, and its pharmacological enhancement is a possible therapeutic goal in neurodegenerative conditions.

## Results

### PrP90-231 induces toxicity of A1 neurons

Misfolded PrP90-231 causes apoptosis in neurons via direct interaction^27,28,51^, or through astrocytic and microglial production of prostaglandins, cyto/chemokines, and nitric oxide^52–54^.

**Fig. 1:**
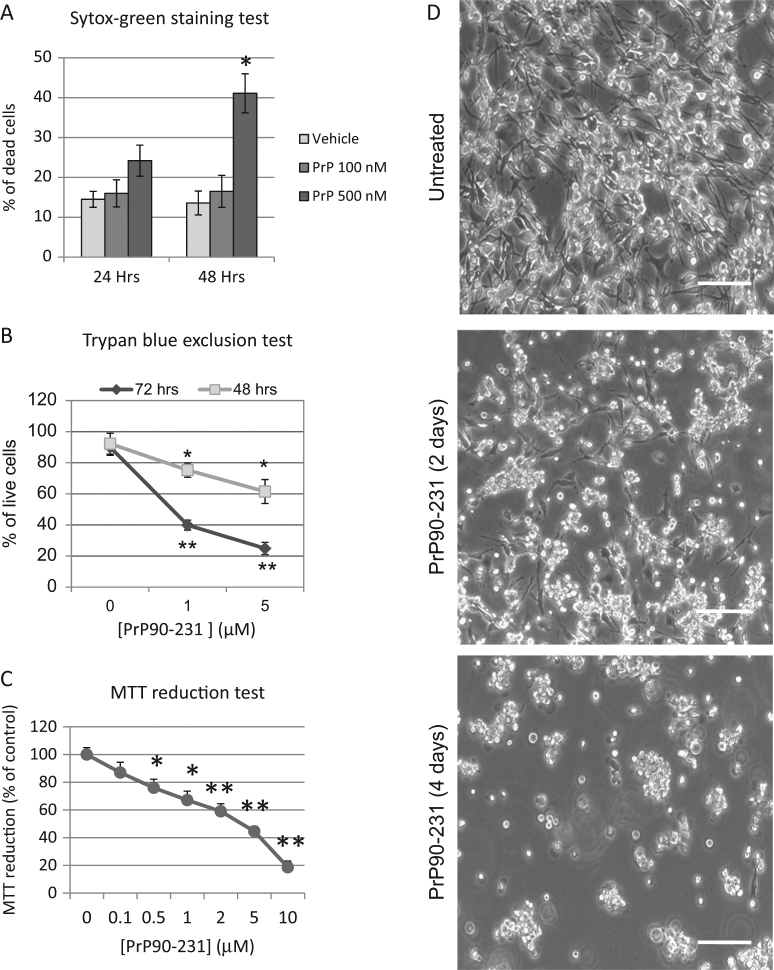
PrP90-231 induces A1 neuron death. **a** Flow cytometry analysis of cell death. Dead cells in the culture were quantified after 24 and 48 h of treatment with PBS (vehicle) or PrP90-231 (100 and 500 nM), by measuring the percent of cells that incorporate SYTOX green. * *P* < 0.05 vs. vehicle. **b** Cell viability was quantified in control cells after 48 and 72 h (treatment with PBS, 0 on *abscissae*) or PrP90-231 (1 and 5 μM) measuring the percent of live cells by Trypan blue exclusion test. *P < 0.05 and **P < 0.01 vs. PBS. **c** Mitochondrial viability of A1 cells was evaluated by MTT-reduction assay after 72 h of treatment with PBS (0 on *abscissae*) and PrP90-231 at concentrations ranging from 0.1 to 10 μM. *P < 0.05 and **P < 0.01 vs. PBS. **d** Phase-contrast pictures of A1 cells that have been treated with PrP90-231 5 μM for two (middle panel) and four (lower panel) days in comparison with untreated (upper panel). Images has been acquired with C- plan 10 × /0.22 magnification lens. Bar 100 μm. All the data are reported as mean + /− SEM of three independent experiments each performed in quadruplicate

Here we used, as neuronal model, A1 immortalized murine mesencephalic neuronal cell line, which combines neuronal phenotype and indefinite in vitro proliferation^54^. SYTOX green staining measured by flow cytometry showed that 48 h PrP90-231 treatment reduced A1 viability up to 41% (Fig. 1a). PrP90-231 neurotoxicity was time- and concentration-dependent, as shown by Trypan blue exclusion test, in which the percentage of surviving cells decreased from 90% (controls) to 75 and 60%, after 48 h exposure to 1 and 5 μM PrP90-231, respectively, and to 40 and 23%, after 72 h of treatment (Fig. 1b). Furthermore, we measured A1 cell viability by MTT assay after 72 h, with PrP90-231 concentrations (0.1–10 μM), which induced a concentration-dependent cell death (Fig. 1c). These results were confirmed by analyzing, by phase-contrast imaging, the A1 cultures after 2 and 4 days of PrP90-231 (5 μM) treatment (Fig. 1d), which shows a diffuse cell damage.

### PrP90-231 is internalized into acidic vesicles

**Fig. 2:**
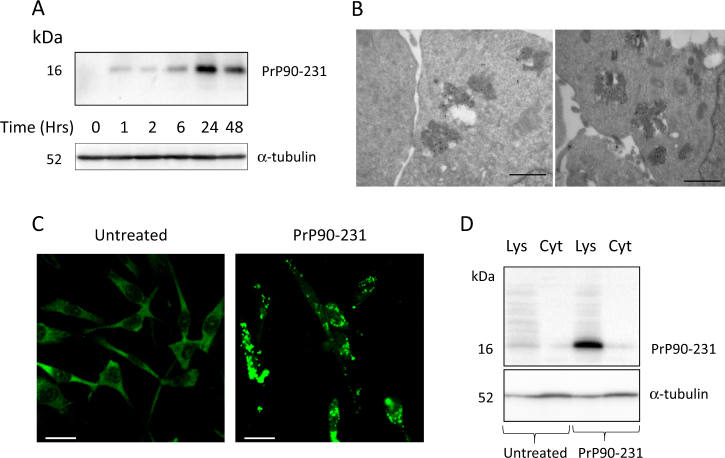
PrP90-231 internalization in A1 neurons. **a** Time-dependent internalization of detergent-insoluble PrP90-231: detergent-insoluble fraction from A1 cells treated for 1, 2, 6, 24, and 48 h with PrP90-231 (1 μM) was extracted and separated by centrifugation; immunoblotting was performed with the 3F4anti-PrP antibody, loading 10 μg protein/lane. Anti-α-tubulin antibody was used to normalize protein loading between lanes. Immunoreactivity for 3F4 antibody revealed a 16 kDa immunoreactive band reaching a maximum intensity after 24 h of treatment with PrP90-231. **b** Immunogold electron microscopy for PrP90-231 intracellular aggregates in A1 neurons treated for 48 h with PrP90-231 (1 μM). Immunogold labeling of anti-PrP antibody FL253 revealed the presence of PrP-immunoreactive signals entrapped into electron-dense vesicles. Space bar 500 nm. **c** Indirect immunostaining with anti-PrP antibody FL235 of A1 cells after 48 h of treatment with PBS (left) or PrP90-231 (1 μM) (right). The exposure to PrP90-231 stimulates the appearance of fl235-immunoreactive spheroidal aggregates; images has been acquired with HCX Fluotar PL 40×/0,75 magnification lens. Bar: 25 μm. **d** Detection by immunoblotting of PrP90-231 in vesicular and cytosolic fraction of A1 cells after 48 h of treatment with PBS or PrP90-231 (1 μM). Immunoreactivity for anti-PrP antibody 3F4 shows the appearance of a 16 kDa band in vesicular fraction only. Immunoreactivity for α-tubulin (lower blot) has also been detected to ascertain that equal amount of protein has been loaded between treated and untreated samples. All the data reported are representative of three independent experiments

To test whether vesicular accumulation of proteolysis-resistant aggregates of misfolded PrP90-231 promotes cell death via impairment of lysosomal stability or represents a neuronal attempt to sequester and eliminate the cytotoxic peptide, we assessed the ability of A1 neurons to internalize PrP90-231 and activate lysosomal/autophagic proteolytic pathways. First, we measured the presence of detergent-insoluble PrP90-231 within A1 cytosol, after 1–48 h of exposure to the peptide. We observed by immunoblotting that PrP90-231 content (16 kDa, 3F4-immunoreactive band, Fig. 2a) gradually increased during the treatment. To localize PrP90-231 within A1 cytoplasmic structures, we evaluated the intracellular accumulation of the peptide by immunogold transmissionelectron microscopy (TEM) (Fig. 2b),  indirect fluorescence  (Fig. 2c), and western blot of lysosomal crude fraction (Fig. 2d). Immunostaining was performed using the polyclonal antibody FL253 that recognizes both endogenous PrP^C^ and PrP90-231, instead of monoclonal 3F4, because the sequence 109-111, specifically recognized by 3F4 antibody might be buried inside PrP90-231 aggregates. In untreated cells, FL253 diffusely stains the cytoplasm without detectable protein clustering (Fig. 2c, left panel), whereas PrP90-231 treatment induced the formation of large aggregates (Fig. 2c, right panel), confirming that PrP90-231 is internalized in subcellular structures, possibly overloading the vesicular compartment. This hypothesis was supported by immunogold/TEM analysis, showing increased number of electron-dense vesicles containing PrP90-231 immunoreactivity (Fig. 2b). Since PrP90-231 amino acid sequence overlaps endogenous PrP^C^ and both are recognized by FL236, to define the source of the immunoreactive clusters observed in PrP90-231-treated cells, we performed a semi-quantitative analysis of PrP90-231 accumulation by immunoblotting, analyzing its distribution between vesicular and cytosolic fractions. Here, we used the 3F4 antibody that does not react with the murine (endogenous) PrP^C^. A 16 kDa 3F4-reactive band was observed only in the vesicular fraction of PrP90-231-treated cells (1 μM, 48 h), but not in the cytosolic fraction (Fig. 2d).

### PrP90-231 triggers lysosomal and mitochondrial destabilization

**Fig. 3:**
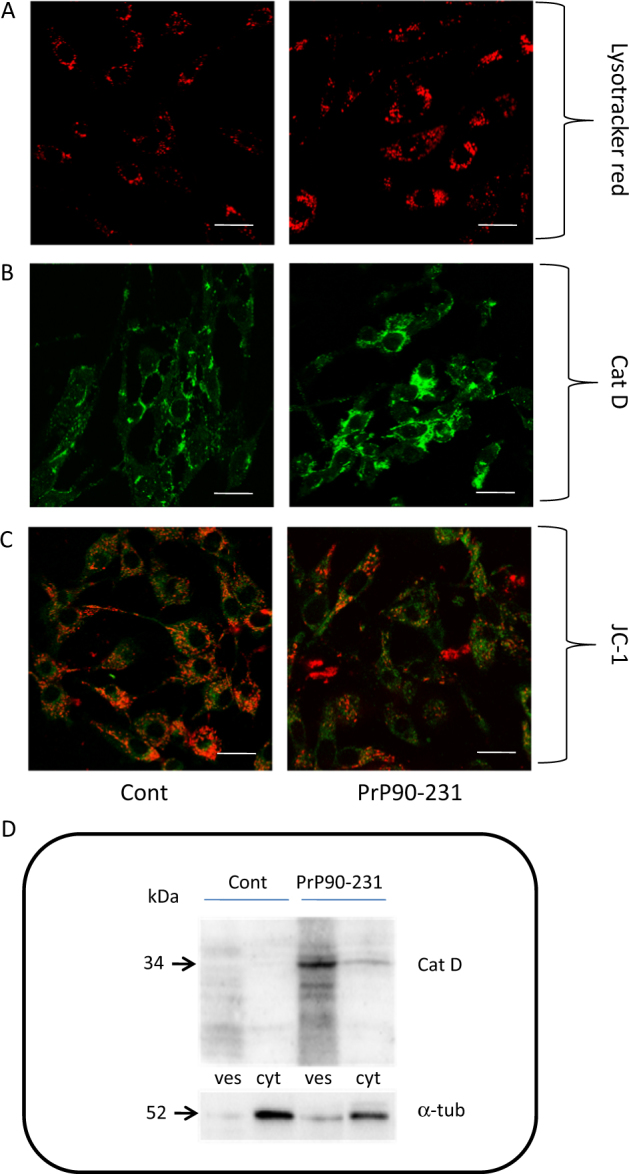
PrP90-231 alters lysosomal permeability and mitochondrial potential. **a** Live cell imaging of acidic vesicles in A1 cells. Cells were grown on glass bottom petri dishes and treated with PBS (cont) and PrP90-231 (1 μM). After 24 h, cells were loaded with acidophilic dye LysoTracker Red DND-22 and observed in confocal microscopy (Scale bar: 25 μm. **b** PrP90-231 increases the expression and cytosolic diffusion of Cat-D in A1 cells. After 48 h of treatment with PBS (cont) and PrP90-231 1 μM, cells were immunostained with anti-Cat-D and observed under inverted fluorescent microscope using magnification lens APO Plan 60 × 1,40 oil. (Scale bar: 25 μm). **c** PrP90-231 triggers A1 mitochondrial depolarization. Cells were grown on glass bottom petri dishes and treated with PBS (cont) and PrP90-231 1 μM. After 24 h, cells were loaded with mitochondrial potential sensor JC-1 and observed in confocal microscopy. The exposure to the peptide induces a red to green shift of JC-1 fluorescence. Scale bar: 25 μm. **d** Detection by immunoblotting of Cat-D expression in vesicular (ves) and cytosolic (cyt) crude fractions in A1 cells after 48 days of treatment with PBS (cont) and PrP90-231 (1 μM). The treatment with PrP90-231 caused a strong increase of Cat-D expression and a detectable diffusion into the cytosolic fraction. All the data reported are representative of three independent experiments

The relevance of lysosomal impairment in PrP90-231 toxicity was analyzed by measuring the effects of PrP90-231 on the expression and cytosol diffusion of Cat-D. We measured, using the confocal microscopy, the number and size of acidic vesicles by A1 neuron labeled with lysotracker red DND-99 (Fig. 3a). While most of the control neurons showed fewer number of lysotracker red-positive vesicles, but after PrP90-231 treatment, a high number of enlarged structures were stained. Since lysotracker red cannot discriminate between lysosomes and autophagosomes, but its fluorescence intensity increases in autophagolysosomes^55^, the above results pose the intriguing question whether the high number of lysotracker red-positive vesicles in treated neurons derived from impairment of autophagic resolution after PrP90-231 accumulation, or it represents the activation of protective mechanisms aimed to eliminate misfolded PrP90-231.

To demonstrate that PrP90-231 causes activation of lysosomal proteolytic activity, by altering lysosomal permeability, we analyzed the expression and the cytoplasmic distribution of the lysosomal protease Cat-D by immunocytofluorescence and immunoblotting. In basal conditions, Cat-D was detectable as small round bodies scattered through the cytoplasm or  localized in the perinuclear region (Fig. 3b), while after PrP90-231 treatment, a strong diffuse Cat-D immunoreactivity was observed throughout the cytosol. Semi-quantitative evaluation of Cat-D expression and localization was performed by immunoblotting in lysosomal and cytosolic crude protein fractions by using antibody that detects the cleaved (active) form of the enzyme (Fig. 3d). Vesicular (lysosomal) and cytosolic crude fractions were separated by differential centrifugation from control and PrP90-231-treated A1. While no significant immunoreactivity was observed in both fractions of control cells, indicating low Cat-D activity, in PrP90-231-treated cells, cleaved Cat-D (37 kDa immunoreactive band) was detected in both lysosomal and cytosolic fractions. Thus the treatment with PrP90-231 induces the activation of Cat-D and causes its cytosolic diffusion. Cat-D diffusion is responsible for mitochondrial depolarization^56^ and initiates caspase-dependent apoptosis. Consequently, we measured mitochondrial membrane depolarization in A1 neurons treated with PrP90-231, using the JC-1 fluorescent probe, which accumulates into mitochondria exhibiting orange/red to green fluorescence shift, upon the loss of mitochondria transmembrane potential. Figure 3c depicts live cell imaging of A1 mitochondria after treatment with vehicle or PrP90-231. PrP90-231 treatment produced a marked reduction of red/orange fluorescence of JC-1, with increasing green-fluorescent mitochondria. Thus, A1 neurons react to PrP90-231 intracellular accumulation with its segregation within lysosomes, resulting in a strong activation of the proteolytic machinery, followed by Cat-D cytosolic diffusion, and mitochondrial destabilization. The persistence of PrP90-231 aggregates within cells and the prolonged activation of hydrolytic enzymes are critical for PrP90-231 to impair the cell survival.

### PrP90-231 impairs autophagy progression in A1 cells

**Fig. 4:**
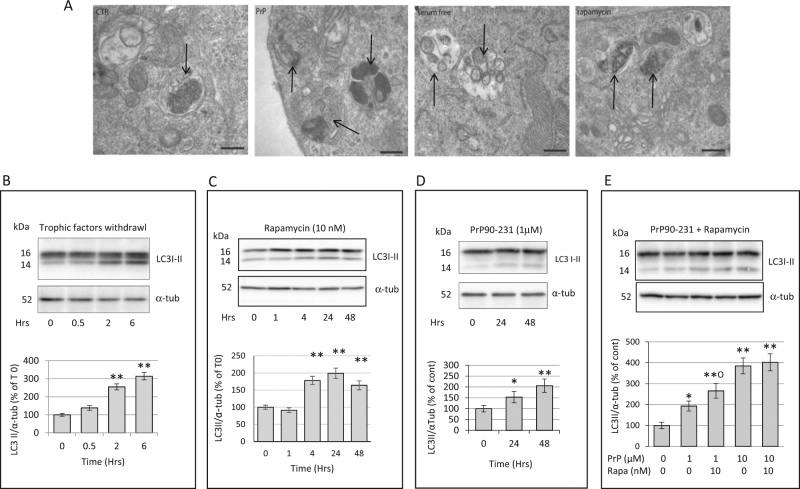
Trophic factor deprivation, rapamycin and PrP90-231 increase the number of autophagosomes. **a** Morphological analysis  of A1 cytoplasm by electron microscopy after trophic factors withdrawal (serum deprivation), treatment with rapamycin 10 nm and PrP90-231 1 µM for 24 h. Images evidenced that serum deprivation and the treatment with both rapamycin and PrP90-231 induced appreciable increase of double-membrane autophagosomes, along with autophagolysosomes, containing electron-dense material (arrows). Space bar: 200 nm. Evaluation by immunoblotting of the time-dependent expression of LC3I-II after trophic factors withdrawal (**b**), rapamycin (10 nM) (**c**), PrP90-231 (1 μM) (**d**), and PrP90-231 (1 and 10 μM) in the absence or the presence of rapamycin (10 nM) (**e**). The amount of autophagosome-bound 14 kDa form of LC3B (LC3BII) was quantified by densitometry (histograms below each blot) and expressed as ratios on α-tubulin expression. LC3BII/α-tubulin values were expressed as percent of time 0 from three separate experiments. PrP90-231, trophic factor withdrawal, and rapamycin induced significant increase in LC3BII. *P < 0.05 and **P < 0.01 vs. time 0. Additive effects of rapamycin on increasing LC3II formation are appreciable only on PrP90-231 (1 μM). **P < 0.01 vs. cont; °*P* < 0.05 vs. PrP90-231. All the data are reported as mean+/− SEM of three independent experiments each performed in quadruplicate

As autophagy is a neuronal survival strategy by removing damaged organelles and degrading protein aggregates, we tested whether lysosome damage by PrP90-231 causes neurotoxicity because of impaired autophagy machinery. A1 neurons are resistant to serum deprivation (data not shown), indicating that these cells are able to activate autophagy-dependent recycle of nutrients, and that the progression of autophagy flux represents a crucial mechanism of protection from cytotoxic insults. We measured autophagy flux in basal conditions, after impairment of cytoplasmic homeostasis, or PrP90-231 treatment (Fig. 4a). The expression of autophagosome-related protein LC3B^57^ was evaluated by immunoblotting in A1 neurons deprived of trophic factors for 0.5–6 h (Fig. 4b) or treated with rapamycin for 2–48 h (Fig. 4c), showing that both conditions increased the accumulation of the 14 KDa form of LC3B (LC3BII), associated with the elongation phase of autophagosomes index of autophagy activation. Also, PrP90-231 treatment (1 μM, for 24–48 h) induced LC3B-II expression, suggesting that intraneuronal accumulation of PrP90-231, besides interfering with lysosomal activity, produces perturbations of  the autophagic flux (Fig. 4d). TEM morphological analysis of A1 cells, performed to assess the presence of autophagy-related organelles after trophic factors withdrawal or rapamycin treatments, evidenced high number of double-membrane autophagosomes that, although present in basal conditions, are significantly increased by all the treatments (Fig. 4a). PrP90-231 (24 h, 1 µM) highly increased the number of autophagic vesicles, although mainly as single-membrane organelles (autophagolysosomes) filled with electron-dense material, suggesting the occurrence of qualitative differences between the autophagic flux induced by the peptide and the other stimuli. LC3B-II immunoblotting performed in A1 neurons after co-treatment with PrP90-231 and rapamycin (Fig. 4e) showed an additive induction of LC3B-II expression mainly in the presence of low PrP90-231 concentration (1 μM) but not when higher concentrations (10 μM) were used, likely due to the saturation of A1 capacity to activate autophagic flow.

**Fig. 5:**
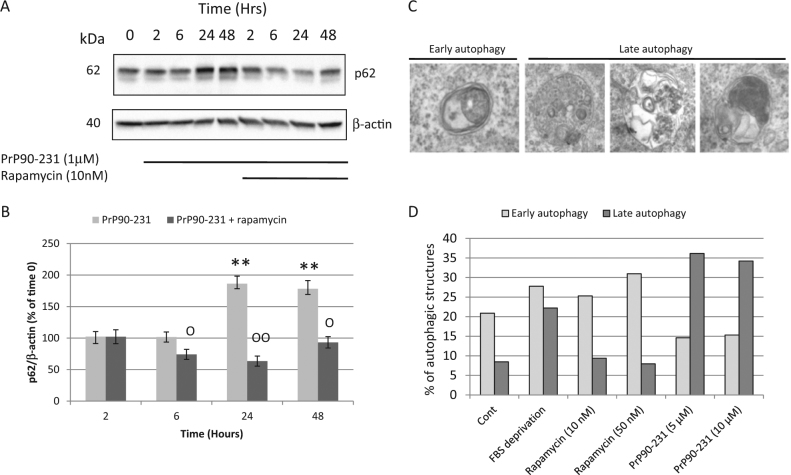
Rapamycin induces autophagosomes formation and autophagy resolution. **a** Cells have been treated for 2, 4, 6, 24, and 48 h with PrP90-231 (1 μM) in the absence or in the presence of rapamycin (10 nM); 50 μg protein/lane from cytoplasmic fractions were resolved by immunoblotting using anti-p62 antibody. **b** P62 immunoreactivity was quantified by densitometry and expressed as ratios on respective β-actin. The treatment with the peptide increases p62 expression; rapamycin strongly reverts PrP90-231 activity on p62. **P < 0.01 vs. time 0; °°*P* < 0.01 vs. time-matching PrP90-231-treated samples. **c**, **d** A1 cells were subjected, for 48 h, to trophic factors withdrawal, or treated with PrP90-231 (5 and 10 µM) or rapamycin (10 and 50 nM), and analyzed by TEM for the presence of different vesicular structures. Panel  **c** shows representative images of double-membrane autophagosomes (early autophagy structures) and single-membrane autophagolysosomes containing partially digested organelles or electron-dense material (late autophagy vesicles). **d** The relative amount of autophagosomes and autophagolysosomes was expressed as percent on all vesicular cytoplasmic structures. All data reported are representative of three independent experiments

LC3B-II expression could be also increased when impaired resolution of autophagolysosomes occurs, as possibly caused by the protease-resistance of PrP90-231. Thus, we investigated whether PrP90-231 treatment interfere with autophagic flux progression, by measuring the expression of the autophagy-adaptor protein p62, which drives cytoplasmic material into autophagosomes and is physiologically digested when autophagosomes fuse with lysosomes^58^. Thus, increased p62 levels are a biochemical hallmark of autophagy blockade^59,60^ and are observed during normal aging or proteinopathies. PrP90-231 treatment time-dependently increased p62 content, with a plateau after 24 h (Fig. 5a,b). These results indicate that autophagy is induced by PrP90-231 but an impairment of the final autophagolysosomes resolution occurs. Importantly, in the presence of rapamycin, p62 increase induced by PrP90-231 was almost completely abolished. Similarly, analyzing the content of autophagy-related vesicles by TEM, we show an inverse ratio between early and late autophagosomes according to the treatments, with the latter that are  accumulated in PrP90-231-treated cells and reduced in the presence of rapamycin (Fig. 5c,d). Thus, we propose that autophagy boost induced by rapamycin can overcome autophagy impairment induced by PrP90-231.

### Activation of autophagy reduces the amount of internalized PrP90-231

**Fig. 6:**
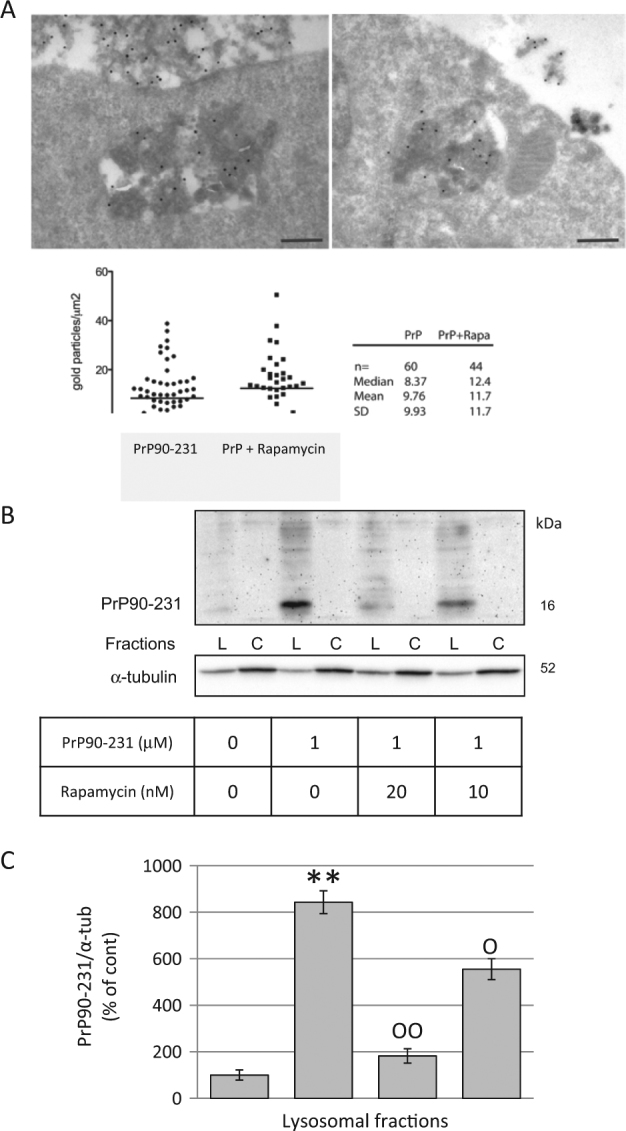
Rapamycin favors autophagy resolution and reduces PrP90-231 intracellular accumulation. **a** A1 neurons treated with PrP90-231 (1 μM, 24 h) in the absence (upper- left panel) or presence (upper-right panel) of rapamycin (10 nM) were subjected to immunogold analysis for PrP90-231 aggregates into electron-dense cytoplasmic vesicles. The number of immunogold-positive spots have been counted and scored as ratio between the number of gold particles and vesicle area (gold particles/μm^2^). **b** After 24 hours of treatment with PBS, PrP90-231 1 mM or PrP90-231 in the presence of rapamycin (10 or 20nM), we separated crude lysosomal (Lys) and cytosolic (Cyt) fractions by differential centrifugation and performed immunoblotting using anti PrP90-231 antibody 3F4. The treatment with PrP90-231 caused a net increase of 16 kDa 3F4-immunioreactive band into the lysosomal fractions that was significantly and concentration dependently reduced by the presence of rapamycin. **c** PrP-immunoreactive bands in lysosomal fraction were quantified by densitometry and expressed as ratios on α-tubulin, from three separate experiments. The treatment with PrP90-231 caused a net increase of 16 kDa 3F4-immunoreactive band into the lysosomal fractions that was significantly and concentration dependently reduced by the presence of rapamycin. **P < 0.01 vs. cont; °*P* < 0.05, and °°*P* < 0.01 vs. PrP90-231. All data reported are representative of three independent experiments.

To test whether rapamycin restores autophagy resolution in PrP90-231-treated cells, we analyze A1 neurons by immunogold TEM for PrP90-231 vesicular accumulation, in the absence or presence of rapamycin (Fig. 6a). Immunogold TEM allowed the determination of the number of autophagic structures containing PrP immunoreactivity (calculated as ratio between the number of immunogold-positive spots and the area of each autophagic structure). We observed that the number of autophagic vesicles containing PrP90-231 were lower in cells co-treated with rapamycin in comparison with neurons receiving PrP90-231 only (Fig. 6a). Furthermore, the amount of PrP90-231 (16 KDa 3F4-immunoreactive band) in the lysosomal fraction was significantly reduced after co-treatment with rapamycin (Fig. 6b,c). These results suggest that the activation of autophagy induced by rapamycin significantly reduces the intracellular accumulation of PrP90-231, likely favoring its clearing by autophagy. Thus, the accumulation of PrP90-231 insoluble aggregates induces autophagic response in neurons but, as its resolution is impaired by insolubility and protease resistance,  it  results in Cat-D diffusion in the cytosol and apoptosis. In the presence of rapamycin, the lower amounts of vesicular PrP90-231 and a complete autophagic flux suggest that proper progression of autophagy causes PrP90-231 digestion, prevents alteration of autophagolysosomal membranes, and favors neuron survival.

### Induction of autophagy protects A1 cells from PrP90-231 toxicity

**Fig. 7:**
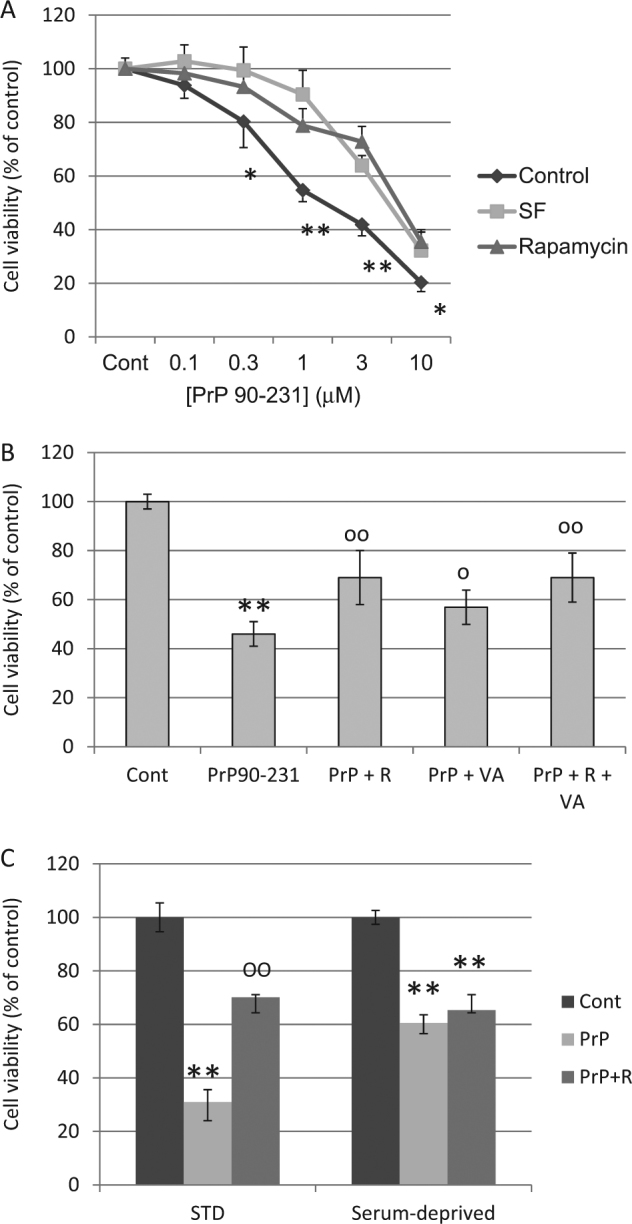
Autophagy enhancement reduces PrP90-231 toxicity on A1 neurons. **a** Concentration-response toxicity of PrP90-231 on A1 cells in standard culture conditions (STD, control) (♦), trophic factors deprivation (SF) (■) or treatment with rapamycin 10 nM (▲); cell viability was measured by MTT assay after 72 h of treatment. Cell culturing in serum-free conditions or treatment with rapamycin reduced significantly PrP90-231 toxicity. * *P* < 0.05; **P < 0.01 vs. SF or rapamycin-treated conditions. **b** Protective activity of valproic acid and rapamycin, against PrP90-231 toxicity. Cells have been treated with rapamycin (10 nM) and valproic acid (1 mM), either alone or in association, before being exposed to PrP90-231 (1 μM) for 72 h. Values of cell viability were determined by MTT assay and reported as percent of untreated samples (Control). Similarly to rapamycin, valproic acid reduced PrP90-231 cytotoxicity, but no additive effects were observed when both compounds were simultaneously added. **P < 0.01 vs. control; °*P* < 0.05, and °°*P* < 0.01 vs. PrP90-231. **c** Lack of additive protection between trophic factors deprivation and rapamycin. Cells were divided in the following two subgroups: (1) grown in standard medium conditions (STD) and (2) deprived of trophic factors for 24 h (SF). Both series were treated for further 72 h with PBS (control), PrP90-231 (1 μM), and PrP90-231 (1 μM) plus rapamycin (10 nM). Cell viability was measured by MTT assay and compared with their respective controls. MTT assay showed that under standard growing conditions (left columns), PrP90-231 exerted significant reduction of cell viability and was strongly antagonized by rapamycin. Conversely, in condition of trophic factors withdrawal, the toxicity of PrP90-231 was less pronounced, although still highly significant, but not affected by treatment with rapamycin. **P < 0.01 vs. control; °°*P* < 0.01 vs. PrP90-231. All data are reported as mean + /− SEM of three independent experiments each performed in quadruplicate

**Fig. 8:**
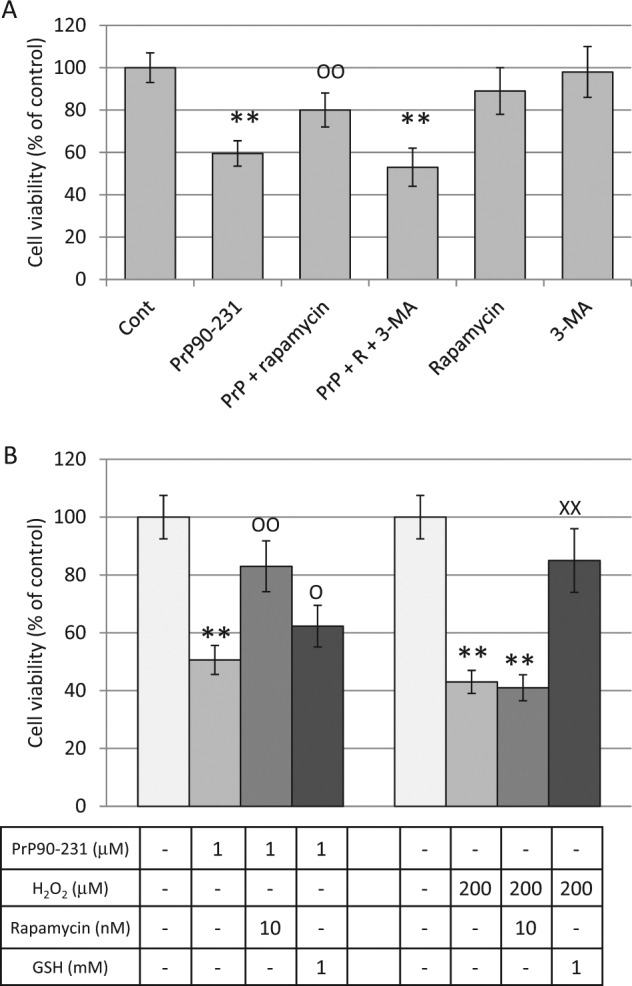
Activation of autophagy as specific mechanism of rapamycin-dependent neuroprotection from PrP90-231 A1 toxicity. **a** Cells were treated with rapamycin (10 nM), 3-methyladenine (3-MA, 1 mM) and PrP90-231 (1 μM); cell viability was measured by MTT assay after 48 h of treatment. The presence of 3-MA fully prevented rapamycin protection of PrP90-231-induced neurotoxicity. **P < 0.01 vs. control; °°*P* < 0.01 vs. PrP90-231. All the data are reported as mean + /− SEM of three independent experiments each performed in quadruplicate. **b** Differential cytoprotection induced by rapamycin and reduced glutathione (GSH) against PrP90-231 and hydrogen peroxide (H_2_O_2_) neurotoxicity. Cells were treated with rapamycin (10 nM) or GSH (1 mM), in the presence of PrP90-231 (1 μM) or H_2_O_2_ (200 μM), for 48 h. GSH was added 1 h before PrP90-231 or H_2_O_2_, since it was reported that is slowly incorporated within the cells^62^. Cell viability was determined by MTT assay, and reported as percent on vehicle-treated samples (Control). PrP90-231 toxicity was strongly reverted by rapamycin, but slightly, although significantly, by GSH; conversely cell death induced by H_2_O_2_ was unmodified by rapamycin and completely abolished by GSH. The data are reported as mean + /− SEM of three independent experiments each performed in quadruplicate. **P < 0.01 vs. control; °*P* < 0.05, and °°*P* < 0.01 vs. PrP90-231; ^XX^*P* < 0.01 vs. H_2_O_2_

To demonstrate that autophagy resolution prevents neurotoxicity, we measured A1 viability after PrP90-231 treatment in conditions that promote autophagy. To elicit physiological or pharmacological activation of autophagy, A1 neurons were deprived from trophic factors for 24 h, or treated with rapamycin or valproic acid before PrP90-231 treatment. Using MTT-reduction assay, we show that the concentration-dependent cytotoxicity of PrP90-231 (0.1–10 μM, for 72 h) was significantly counteracted, although not abolished in serum-deprived or rapamycin-treated A1 neurons (Fig. 7a). Noteworthy, the protective effect of serum withdrawal and rapamycin were dependent on PrP90-231 concentration, being highly significant for concentration between 1 and 3 μM, and less effective for lower (0.1–0.3 μM) or higher (10 μM) concentrations. Similarly, autophagy activation by valproic acid (1 mM)^61^ antagonized PrP90-231 neurotoxicity (Fig. 7b). To demonstrate that rapamycin neuroprotection is dependent on autophagy enhancement, we tested its activity in experimental conditions in which autophagy was already induced. First, we tested whether rapamycin and valproic acid protection was additive, showing that the combination of the two drugs matched perfectly the effects induced by the individual agents (Fig. 7b). Then, A1 neurons were exposed to PrP90-231, with or without rapamycin, after 24 h of growth in standard medium (STD) or trophic factor withdrawal (serum-deprived cells); cell viability was evaluated after further 48 h (Fig. 7c). PrP90-231 neurotoxicity was reduced in cells grown in conditions of nutrient deprivation (Fig. 7a), but not further protection was observed in the presence of rapamycin (Fig. 7c), which, otherwise, antagonized PrP90-231 in standard culture conditions (Fig. 7c, left columns). Moreover, rapamycin protection against PrP90-231 neurotoxicity was abolished by autophagy inhibition induced by 3-methyladenine (3-MA)^57^ (Fig. 8a). Conversely, the blockade of autophagic response by 3-MA did not affect PrP90-231 toxicity (not shown), confirming that the increased autophagy occurring in these conditions was not causally related to PrP90-231 neurotoxicity but represents a tentative to eliminate the misfolded peptide. Thus, rapamycin does not induce additive  neuroprotection when autophagy is already activated (as occurs after valproic acid addition or growth factor starvation), while its effects are abolished when autophagy is pharmacologically blocked. Conversely, when A1 neurotoxicity was induced through different pathways such as oxidative stress caused by hydrogen peroxide (H_2_O_2_, 200 μM), rapamycin was completely ineffective, while neuronal death was prevented in the presence of the antioxidant reduced glutathione (GSH)^62^ (Fig. 8b). Similarly, rapamycin did not protect from cell death induced by nitric oxide radicals (sodium nitroprussiate treatment) or by excitotoxicty (by glutamic acid) (data not shown). These data suggest that neuroprotective effects of rapamycin are mediated only by increasing autophagy, mainly when this mechanism is impaired by misfolded peptides, without significant autophagy-independent effects. However, while the activation of autophagy by rapamycin, valproic acid, or serum deprivation is the main mechanism of protection against PrP90-231 neurotoxicity, these treatments did not completely abolish PrP90-231-dependent cell death, suggesting that different autophagy-insensitive mechanisms are involved in PrP90-231 neurotoxicity, which, in part, might involve ROS accumulation caused by mitochondrial dysfunction. In fact, GSH treatment, slightly, but statistically significantly, protected A1 neurons from PrP90-231 toxicity (Fig. 8b), indicating that ROS accumulation co-participate to the neurotoxicity of the prion peptide.

## Discussion

Efforts to develop therapies against TSE mainly focused on preventing PrP^C^=>PrP^Sc^ conversion and the spreading of PrP^Sc^ aggregates. Several molecules, including the anti-malarial quinacrine and tetracycline antibiotics, have been tested to prevent the formation, or destabilize PrP^C^–PrP^Sc^ complex^63–66^. The outcomes have been so far frustrating because molecules that prevented prion infectivity in vitro or delayed clinical onset in experimentally ill animals failed in humans^67,68^. Although less challenged, the prevention of PrP^Sc^ neurotoxicity is a further pharmacological strategy for TSE and other neurodegenerative disorders^54,69^. Indeed, all proteinopathies are characterized by aggregation of altered proteins responsible for neurodegeneration through the formation of neurotoxic species along with the process of misfolding independently from the amino acid sequence^3^.

Brain deposition of amyloid proteins often associated with significantly increased autophagy activity, and neurodegeneration is aggravated by conditions that impair the progression of autophagic flux in neurons, suggesting that autophagy could represent a mechanism involved in neuronal loss in neurodegenerative disorders^39–41^. On the other hand, pharmacological enhancement of autophagy can inhibit prion neurotoxicity and infectivity in vitro, and reduce pathogenicity in animal models of TSE^38,70,71^, indicating that boosting cellular mechanisms devoted to protein clearance provides neuroprotection.

The present work originates from the observation that intracellular accumulation of neurotoxic PrP90-231 produces the formation of insoluble and protease-resistant aggregates associated with impairment of lysosome membrane permeability^51^. Here, we determined the nature of these cytoplasmic inclusions and characterized the role of lysosomes and autophagy activation as cell response to PrP90-231 toxicity. PrP90-231 sequence represents the in vitro counterpart of the amyloidogenic PrP^Sc^ core^44^ that coexists with full-length protein in TSE brains, and associates with prion infectivity^72^ and neurotoxicity^73^. PrP90-231 relevance, as model of neurotoxicity, resides in  the possibility of refold it into a β-sheet-rich conformation to increases aggregation propensity^47^. During the aggregation process, soluble oligomers are formed as intermediate products^50^, reproducing structural and neurotoxic features of oligomers responsible of other neurodegenerative diseases^22^. As a neuronal model, we used the immortalized cell line A1 obtained from mesencephalic embryonic neurons^74^, which displayed significant sensitivity to PrP90-231, different from the SH-SY5Y neuroblastoma cells that was used in our previous study^51^, and were able to survive in conditions of deprivation of nutrients (i.e., fetal bovine serum) up to 6 days. Such resistance, likely sustained by efficient recycling of intracellular elements through autophagy, indicates that A1 neurons are a suitable cell model to investigate the role of proteostasis machinery in modulating the toxicity of misfolded proteins. TEM analysis supported this hypothesis since a significant increase in vesicular structures, belonging to autophagy flux after cell starvation or treatment with rapamycin was observed. Moreover, analysis of the expression of autophagosome-related proteins LC3II produced consonant results, indicating that autophagosomes content is regulated by starvation or pharmacological disinhibition. In basal conditions, A1 neurons showed significant sensitivity to PrP90-231 neurotoxicity, with cell death time-dependently associated with peptide internalization, increased expression and cytosolic diffusion of the active Cat-D, and reduction of mitochondrial membrane potential. Being PrP90-231 partially resistant to proteolysis, its internalization, followed by aggregation within lysosomes may impair their activity and membrane impermeability, turning the increase of proteolytic activity from a beneficial strategy for proteostasis into a proapoptotic mechanism^51,75^. In this context, the progressive increase of the autophagosome-linked p62 protein during PrP90-231 treatment shows that the intracellular aggregation of the peptide stimulates autophagic flux, but leads to the accumulation of autophagolysomes with impaired resolution ability. Rapamycin co-treatment prevents PrP90-231-dependent p62 accumulation, indicating that the incomplete autophagy induced by the peptide can be pharmacologically unlocked.

In glioma cells, PrP^C^ sustains cell viability by preventing induction of autophagy, since its gene silencing determine autophagy-dependent apoptosis^76^. Thus, altogether, these data support the notion that, according to cell type and physiological status, both inhibition and induction of autophagy can cause cell death and, therefore, autophagy is a main pharmacological target to control cell viability. The main result of our study is that different treatments causing mTOR inhibition strongly reduce PrP90-231 toxicity, suggesting that boosting autophagy promotes degradation of intralysosomal PrP90-231, preventing cytosolic diffusion of lysosomal enzymes from damaged membranes. Importantly, since it was demonstrated that autophagy may be neuroprotective by clearing cytoplasm from damaged lysosomes or mitochondria^77,78^, it is also reasonable that nutrient deprivation or rapamycin protect neurons acting downstream to PrP90-231 accumulation, removing lysosomes filled with PrP90-231. Although we have no clear evidence of lysosomal or mitochondrial entrapment into autophagosomes, such intriguing possibility will be further addressed. Importantly, rapamycin-protective effects were solely mediated by the activation of autophagy, since the same treatment did not protect cells from other toxic stimuli (oxygen or nitric oxide radicals, excitotoxicity), remarking the relevance of the control of autophagy flux to prevent misfolded protein-related neuronal death. Importantly, pharmacological protection from PrP90-231 neurotoxicity has been obtained using drugs, rapamycin and valproic acid, currently used in human therapy, and thus beyond being proof-of-principle tools, they represent a feasible therapeutic approach to be tested in clinical trials for TSE and, possibly, for other proteinopathies.

## Materials and methods

### Antibodies and chemicals

#### Antibodies

Mouse monoclonal anti-PrP antibody (clone 3F4) was purchased from Signet Lab, London, UK. Rabbit polyclonal anti-PrP (clone FL 253) and anti-cathepsin-D (active fragment) were purchased from SantaCruz, CA, USA. Anti-LC3BI-II and anti-P62 were purchased from Cell Signaling Technology. Anti-β-actin and anti-α-tubulin mouse monoclonal antibodies were from Sigma-Aldrich, Milano, Italy.

#### Dyes

LysoTracker^TM^ Red DND-99 and JC-1 were from purchased Molecular Probes, Thermofisher Scientific.

#### Chemicals

Rapamycin was purchased from Cell Signaling Technology; 3-methyladenine, valproic acid, and reduced glutathione (GSH) from Sigma-Aldrich, Italy.

### Synthesis and refolding of PrP90-231

PrP90-231 was obtained from transformed *E. coli* and purified as previously described^79^. To induce structural refolding, the protein was incubated for 1 h in NaCl-free 10 mM phosphate buffer, pH 7.2 at 53 °C^47^. This controlled thermal denaturation protocol induces a three-dimensional refolding of PrP90-231 into a β-sheet-rich structure, inducing gain of toxicity in vitro^47^. Thereafter, the term “PrP90-231” will refer to thermally-refolded peptide throughout the paper. Cell treatments were performed adding the refolded recombinant peptide directly to the culture medium.

### Cell cultures

Murine mes-c-myc A1 (A1) neurons^74^ were cultured with RPMI medium (EuroClone, Pero (MI), Italy) supplemented with 100 U/ml penicillin, 100 μg/ml streptomycin, 2 mM glutamine (EuroClone, Pero, Milano, Italy), 5% fetal bovine serum (Gibco-BRL, Milan, Italy), and maintained at 37 °C in humidified 5% CO_2_ atmosphere.

### Evaluation of cell viability

#### Phase-contrast microscopy

A1 cells were observed under phase contrast and photographed using a Leica microscope DM IL, equipped with ICC50 HD camera.

#### SYTOX staining

This assay employs the property of green-fluorescent dye SYTOX green to cross the plasmamembrane and stain the nucleic acid of dead cells. Briefly, A1 cells were plated on 12-well cluster dishes at the concentration of 50*10^3^/well and treated with PrP90-231 (100 and 500 nM) for 48 h. After 15 min of dark-adapted incubation with SYTOX (ThermoFisher Scientific), 100 nM at r.t., cells were collected and processed into Cyan ADP Cytofluorimeter (Beckman-Coulter, Brea, CA, USA) using a 488/530 excitation/emission setting; the data were analyzed with Summit 4.3.1 software (Dakocytomation, Ely, Cambridgeshire, UK).

#### MTT reduction assay

Mitochondrial dehydrogenase activity, as index of cell viability was evaluated measuring the conversion of water-soluble (3-(4, 5-dimethylthiazol-2-yl)-2,5)-diphenyltetrazolium bromide (MTT, Sigma-Aldrich, Milano, Italy) into purple, water-insoluble formazan crystals. MTT (0.25 mg/ml) was added into culture medium and incubated for 2 h at 37 °C^80^. Formazan crystals were solubilized with dimethylsulfoxide and the concentration was measured with ELx800 BioTek colorimeter (optical density 570 nm).

#### Trypan blue exclusion test

Cell viability was deduced from the number of Trypan blue-impermeant cells. Briefly, cells were collected in phosphate-buffered saline (PBS), pelleted by microcentrifugation, and resuspended in serum-free RPMI growth medium. Cell suspensions were mixed with one volume of 0.4% Trypan blue solution and immediately analyzed using the automated cell counter TC20 (Bio-Rad)^54^.

### Analysis of protein expression

#### Immunostaining

Cells were seeded in 13 mm glass coverslips at 50% confluence and allowed to grow for 24 h before being treated according to the experimental protocol. After treatments, cells were rinsed with PBS, fixed with ice-cold methanol for 10 min, and permeabilized with PBS containing 0,1% triton X-100 for 30 s at r.t. Fixed cells were then incubated for 15 min. at r.t. in PBS containing 2% normal goat serum (NGS) to block nonspecific sites and probed with primary antibodies diluted in PBS–NGS 2% for 1 h at r.t.^81^. Immunoreactivity was evidenced by incubating the coverslips with antisera conjugated with AlexaFluor 564 (1 h r.t.). Fluorescence was visualized under fluorescence microscope Leica DM2500 equipped with Leica DFC310FX camera.

#### Total cytoplasmic protein extraction

Cells collected in lysis buffer containing TRIS (20 mM, pH 8), NaCl (137 mM), EDTA (2 mM), glycerol (10%), NP40 (1%), Na orthovanadate (1 mM), phenylmethylsunfonyl fluoride (PMSF, 1 mM), and the “Complete” protease inhibitor cocktail (Roche) were cleared of nuclei and debris by centrifugation to yield cytoplasmic fractions; protein concentration was measured by Bradford assay to load equal amounts of proteins in each sample.

#### Cytoplasm fractionation

Cells were collected in buffer containing sucrose (250 mM), EDTA (1 mM), HEPES (10 mM pH 7.4), PMSF (1 mM), and the “Complete” protease inhibitor cocktail (Roche), and homogenized with Teflon-glass potter (Wheaton Scientific USA) (30 strokes at 4 °C). Protein concentration was measured by Bradford assay and samples containing 1 mg/ml of total proteins were subjected to centrifugation at 500 rcf for 10 min to clear cytoplasmic content from nuclei. Lysosomal crude fractions were separated from microsomal and cytosolic proteins by centrifugation at 20,000 rcf for 20 min.

#### Immunoblotting

Samples containing equal amounts of proteins were boiled in Laemmli denaturing buffer, size-fractionated by SDS-PAGE, and transferred on PVDF 0.2 μm membrane (Bio-Rad). Membranes were blocked with skim milk (5% w/vol) in TRIS-buffered saline containing 0.1% tween-20 (TBS-tween), and probed overnight with primary antibodies (diluted in TBS-Tween). Immunoreactivity was detected with horseradish peroxidase-liked antisera (GE-Healthcare) and revealed by enhanced chemiluminescence (ECL, Bio-Rad)^82^. Protein expression levels were quantified by densitometric analysis of immunoreactivity, using the Chemidoc XRS apparatus (Bio-Rad Laboratories).

### Live cell imaging

Cells were seeded in 35 mm glass bottom petri dishes (IWAKI) at 50% confluence, and allowed to grow for 24 h before being treated according to experimental protocol. After treatments, cells were loaded with viable fluorescent probes and visualized under inverted fluorescence microscope (Nikon) equipped with confocal laser scanning system (Bio-Rad MRC1024ES).

#### Acidic vesicle imaging

Treated cells were incubated with LysoTracker^TM^ Red DND-99 (50 nM at 37 °C in complete medium) for 15 min. Cells were then washed twice with medium to remove excess dye, and observed under confocal microscopy using optical settings for rhodamine (564/590).

#### Analysis of mitochondrial potential

The viable dye JC-1 exhibits selective tropism for mitochondria and Abs/Em spectrum that shifts from red to green along with the decrease of mitochondrial potential, thus indicating early apoptosis^56^. Briefly, after treatments with PrP90-231, cells were incubated with mitochondrial potential sensor JC-1 (10 μg/ml at 37 °C in complete medium). After 10 min, cells were washed twice with medium, to remove excess of JC-1, and observed under confocal microscopy. Optical settings for rhodamine (564/590) and fluorescein (488/530) were used.

### Transmission electron microscopy (TEM)

A1 cells were seeded in glass chamberslides (Lab-Tek, Nunc, 177380) for 48 h at 37 °C and treated for further 48 h with serum-free medium, PrP90-231 (1 µM), and PrP90-231 (1µM) with rapamycin (10 nM). Cells were washed with 0.1 M cacodylate buffer in distilled water and fixed in the same buffer containing 2.5% glutaraldehyde for 1 h at r.t. Cells were postfixed in 1% osmium tetroxide for 10 min and 1% uranyl acetate for 1 h, dehydrated through a graded ethanol series embedded in epoxy resin (Poly-Bed; Polysciences, Inc, Washington, PA, USA) overnight at 60 °C. Ultrathin sections (50 nm) were observed with a CM10 (Philips, Eindhoven, The Nederlands) without additional staining. Digital images were taken with Megaview 3 CCd camera and iTEM software and processed with Adobe Photoshop CS5.

### Immunogold labeling on Tokuyasu cryosections

A1 cells were treated with PrP90-231 (10 µM) with or without rapamycin (10 nM) for 48 h and then prepared for cryoimmuno-EM according to Tokuyasu method. Briefly, cells were fixed in PBS containing 2% paraformaldehyde and 0.2% glutaraldehyde, for 2 h at rt. Next, cells were gently scraped and embedded in 12% gelatin. After overnight infusion with sucrose 2.3 M, small squared blocks were mounted on aluminum pins and frozen in liquid nitrogen. Ultrathin sections of 60 nm were cut with Leica UltraCut UCT microtome (Leica Mycrosystems, Wetzlar, Germany) and immunolabeled with the polyclonal anti-prion antibody FL253. Protein A-gold 15 nm was used to reveal prion protein in extracellular and intracellular compartments. Gold particles representing internalized PrP90-231 in intracellular vesicular compartments were counted in 10 cellular profiles.

### Statistics

The data from cell viability assays and immunoblotting densitometries were obtained, respectively, from three independent experiments performed in quadruplicate, unless otherwise specified. Values were expressed as mean ± S.E.M. Statistical differences among values (calculated with one-way ANOVA/GraphPad Prism 5.2) were considered significant and highly significant for *P* values ≤ 0.05.
